# Function of the tryptophan metabolite, L-kynurenine, in human corneal endothelial cells

**Published:** 2009-07-08

**Authors:** Nermin Serbecic, Imad Lahdou, Alexander Scheuerle, Romana Höftberger, Fahmy Aboul-Enein

**Affiliations:** 1 Department of Ophthalmology, Medical University of Vienna, Vienna, Austria; 2 Institute of Immunology, Department of Transplantation Immunology, University of Heidelberg, Heidelberg, Germany; 3 University Eye Hospital Heidelberg, INF400, Heidelberg, Germany; 4 Institute of Neuropathology, Medical University of Vienna, Vienna, Austria; 5 SMZ-Ost Donauspital, Department of Neurology, Vienna, Austria

## Abstract

**Purpose:**

Penetrating keratoplasty has been the mainstay for the treatment of blindness and is the most common form of tissue transplantation worldwide. Due to significant rates of rejection, treatment of immunological transplant reactions is of wide interest. Recently in a mouse model, the overexpression of indoeleamine 2,3 dioxigenase (IDO) was led to an extension in corneal allograft survival. L-kynurenine is a tryptophan metabolite, which may render activated T-cells apoptotic and therefore might modulate an allogenous transplant reaction. The function of L-kynurenine in the human cornea remains unclear. We analyzed the expression levels of IDO in human corneal endothelial cells (HCECs) and downstream tryptophan/kynurenine mechanisms in cell culture.

**Methods:**

An immunological activation profile was determined in proliferation assays of monocytes from healthy donors. Reversed-phase high pressure liquid chromatography (HPLC), western blot, real time polymerase chain reaction (PCR), and microarray analyses were used. The expression of IDO and immunological infiltration of rejected human corneal allografts (n=12) were analyzed by immunohistochemistry.

**Results:**

We found IDO and an associated tryptophan/kynurenine transporter protein exchange mechanism upregulated by inflammatory cytokines in HCECs. The inhibition of T-cell proliferation might depend on rapid delivery of the tryptophan metabolite, L-kynurenine, to the local corneal environment. Microarray analysis gives evidence that the large amino acid transporter 1 (LAT1) transporter protein is responsible for this mechanism.

**Conclusions:**

Our data support that adequate levels of functional L-kynurenine might contribute to the maintenance of a relative immune privilege in the ocular anterior chamber, thereby contributing to the preservation of corneal allogeneic cells.

## Introduction

With over 60,000 procedures performed worldwide each year, corneal transplantation is the most common form of solid tissue engraftment. Penetrating keratoplasty (PKP) has been the mainstay of treatment for blindness due to corneal diseases. Keratoconus, inherited disorders, scarring caused by infections (herpes simplex virus), trauma or chemical injury, and opacification following cataract surgery [1-3] are many examples of clinical indications for corneal transplantation. Despite being an immune privileged site, all reports indicate that immunological rejection remains the leading barrier to long-term corneal graft survival (10–15 years). Significant portion of corneal failure is due to allogeneic rejection [4]. Although rejection can occur in any of the three corneal layers (epithelium, stroma, or endothelium), the single layered endothelium is the major contributor toward allograft failure the majority of time [5]. In addition, the natural decrease of human corneal endothelial cells (HCECs; >4,000 HCECs/mm^2^ at birth versus 2,500 HCECs/mm^2^ in adulthood) is further exacerbated by the loss of HCECs as a consequence of cell apoptosis during ex vivo storage of corneal grafts or following the transplant procedure itself or early postoperative inflammation. Other factors such as a slow-rate of endothelial attrition (~4% a year) that are not associated with any overt rejection episodes can also confound the loss of endothelial layer function [4].

Due to the nonregenerative capacity of the endothelial layer, its loss due to an alloimmune attack can often lead to blindness when the density of endothelial cells falls below a critical value of 500 cells/mm^2^ [4,6]. It is therefore essential for nature to develop a specific mechanism to allow for appropriate corneal protection from immunological insults. Endothelial apoptosis might take place, especially in an environment with high nitric oxide [7]. Understanding the mechanism will ultimately afford to develop a novel strategy to combat the allorejection in clinical transplantation. One of recently identified pathways to modulate T-cell response during allogeneic transplant rejection is through an immunosuppressive enzyme, indoleamine 2,3-dioxygenase (IDO), which degrades tryptophan into kynurenine. IDO can be induced by interferon gamma (IFN-γ) in various cell types including vascular or corneal endothelial cells [8,9]. However, the exact mechanism by which tryptophan degradation in the anterior chamber mediates its potential tolerogenic effects has yet to be defined. Overexpression of IDO can prolong allograft survival of various organs including murine corneas [8,10,11].

The purpose of our study was to investigate IDO expression in human corneal endothelial cells (HCECs) and the functional role of the tryptophan/kynurenine downstream pathway on allogeneic T-cell proliferation and HCEC survival.

We compared the expression levels of IDO in HCECs and their biological function, namely the L-kynurenine levels, to inactivate TNF-α dependent T-cell responses. Consequently, we identified a tryptophan/kynurenine exchange mechanism, which is upregulated in HCECs by inflammatory cytokines. We propose that adequate L-kynurenine levels might be important in the maintenance of a relative immune privilege in the ocular anterior chamber.

## Methods

### Cell culture

The human corneal endothelial cell line was obtained from the Hamburg Eye Bank, University of Hamburg, Department of Ophthalmology, Hamburg, Germany (Dr. J. Bednarz) as previously published [12]. The cells were grown in RPMI 1640 (Promocell, Heidelberg, Germany) supplemented with 10% heat-inactivated fetal bovine serum (Promocell), 1% L-glutamine (Promocell), 1% penicillin/streptomycin mix (Promocell) in six-well plates (Cellstar; Greiner Bio-One, Essen, Germany). In some cases, the cells were stimulated with either interferon gamma (IFN-γ) and/or tumor necrosis factor alpha (TNF-α). Prior to stimulation, HCECs were grown to a density of 5.0×10^5^ cells per well. They were then stimulated in a humidified atmosphere of 5% CO_2_ for three days in six-well plates at the following concentrations: 500 ng/ml IFN-γ, 100 units/ml TNF-α, or 500 ng/ml IFN-γ together with 100 units/ml TNF-α.

### High performance liquid chromatography

Culture supernatants were harvested to quantify the concentration of L-tryptophan and L-kynurenine using reversed-phase high pressure liquid chromatography (HPLC) as described previously [13]. The samples were deproteinized with 2.4 M perchloric acid for 5 min. After centrifugation (1,000x g for 15 min at 4 °C), supernatants were transferred into new tubes. The pH value adjusted to 7.0, and the 100 µl filtered supernatant was injected into a C-18 column (Supelco, Aschaffenburg, Germany). Samples were eluted with PBS over 30 min. Tryptophan was monitored by means of its native fluorescence at the excitation wavelength of 285 nm and emission wavelength of 360 nm whereas L-kynurenine was detected simultaneously by ultraviolet (UV) absorption at the wavelength of 230 nm. The peaks of L-kynurenine and L-tryptophan were identified following their comparison with the retention time of previously determined standard compounds. Quantification was based on the ratios of the peak areas of the compound to the internal standard [13].

To determine whether the decrease of L-tryptophan and the increase of L-kynurenine were caused by IDO, we probed for this enzyme on protein and mRNA levels.

### Electrophoresis and quantitative immunoblotting assay

Cell lysates were prepared by re-suspending 1×10^6^ HCECs first in a 250 µl lysis buffer (PBS, 1% Nonidet P-40, 0.5% sodium deoxycholate, 0.1% SDS, 100 ng/ml PMSF, 66 ng/ml aprotinin). Total proteins (50 µg/sample) were boiled for 10 min at 95 °C. Protein samples were separated on 10% SDS-polyacrylamide gel (BioCat, Heidelberg, Germany) and then transferred to polyvinylidene difluoride membranes (Immobilon Transfer Membrane; Millipore, Schwalbach, Germany) using standard electrophoretic transfer methods. To prevent nonspecific binding, the membranes were incubated with blocking buffer according to the kit instructions (WesternBreeze® WB7104, WB7106, WB7108; Invitrogen, Karlsruhe, Germany) and then incubated with IDO monoclonal antibody (MAB5412; Chemicon, Hofheim, Germany) at room temperature for 1 h.

This was followed by a washing step and the incubation of the blots with the secondary antibody with Alexa Fluor 680 goat anti-mouse IgG(H^+^L) at room temperature for 1 h (Invitrogen, Karlsruhe, Germany). Bands were visualized detected using the Li-Cor-Odyssey scanner; Li-Cor, Bad Homburg, Germany. Quantization was performed using the Odyssey image software (Li-Cor). Bands were quantified using a dilution series of human recombinant IDO protein (0.3 µg, 1.5 µg, and 3.0 µg) as described above [14]. Recombinant IDO protein was purchased from Millipore.

For the detection of large amino acid transporter 1 (LAT1) protein, cell lysates were generated as described above. The cells have been used in the following conditions: unstimulated HCECs, HCECs stimulated with IFN-γ, HCECs stimulated with IFN-γ+TNF-α, and HCECs stimulated with TNF-α. As a positive control, we used the liver cell line, HUH-7, as previously described [15]. The primary incubation was performed using the rabbit-anti-human antibody directed against LAT1 (AbD Serotec, Duesseldorf, Germany) for 1 h at room temperature. This was followed by a washing step and the incubation of the blots with the secondary antibody with Alexa Fluor 680 goat anti-rabbit IgG (Invitrogen) at room temperature for 1 h. Bands were visualized as described above.

### RNA extraction and quantitative real-time RT-PCR

**Figure 1:**
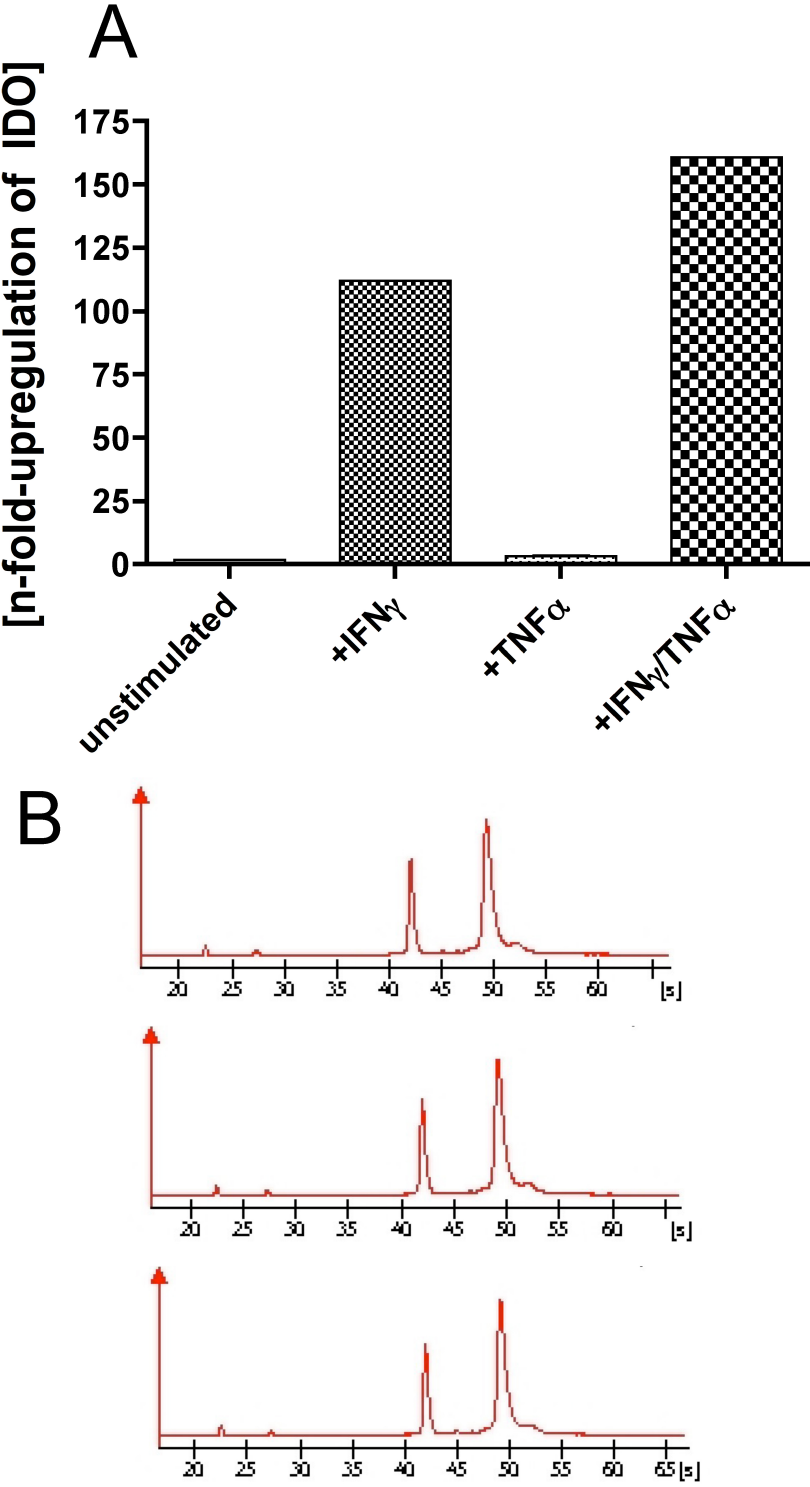
*IDO* mRNA was quantified by real-time PCR. **A**: Products show the results from RNA extracted from four pooled corneal endothelial cell cultures. *IDO* mRNA levels are normalized to *GAPDH*. Higher levels of *IDO* mRNA expression are seen in pools of HCECs that have been stimulated with 500 ng/ml IFN-γ and 500 ng/ml IFN-γ+100 U/ml TNF-α for three days. The y-axis indicates *IDO* mRNA fold change. The x-axis presents the difference in four pool treatments. Lane 1, unstimulated; lane 2, 500 ng/ml IFN-γ; lane 3, 100 U/ml TNF-α; lane 4, 500 ng/ml IFN-γ + 100 U/ml TNF-α. **B**: The bottom panels show examples of the determination of RNA using the Bioanalyzer: unstimulated HCECs (top), IFN-γ treated HCECs (middle), and IFN-γ/TNF-α treated HCECs (bottom).

Total RNA was isolated using RNeasy-plus Mini Kit spin columns (Qiagen Gmbh, Hilden, Germany). Quantification of total RNA was performed in a NanoDrop (ND-1000; NanoDrop Technologies, Wilmington, DE) spectrophotometer. The quality of the RNA obtained was proved using the RNA 6000 Nano Assay on an Agilent 2100 Bioanalyzer (Agilent Technologies, Palo Alto, CA). All samples were used only at the highest quality (Figure 1B).

One microgram of total RNA was used for cDNA synthesis by reverse transcription. First strand synthesis was performed with the SuperScriptIII, First-Strand Synthesis SuperMix for quantitative reverse transcription polymerase chain reaction (qRT–PCR) module (Invitrogen). The protocol provided by the enzyme manufacturer (Invitrogen) was strictly followed except in the omission of DTT, which was found to reduce the efficiency of real-time PCR [16].

The real-time PCR analysis was performed with the SYBR GreenER Two-Step Qrt-PCR Kit Universal (Invitrogen) on an Applied Biosystems-7500 real-time PCR machine (Applied Bioscience, Darmstadt, Germany). Glycerinaldehyde-3-phosphate-dehydrogenase (*GAPDH*) was used as an endogenous control gene to normalize for varying starting amounts of RNA. The following oligonucleotides (MWG Biotech) were used: *GAPDH* sense primer 5′-GAA GGT GAA GGT CGG AGT-3′ and the antisense primer 5′-AGA TGG TGA TGG GAT TTC-3′ and the *IDO-1* sense primer 5′-CAG CTG CTT CTG CAA TCA AA-3′ and the antisense primer 5′-AGC GCC TTT AGC AAA GTG TC-3′. The instrument settings were as follows: initial denaturation step of 10 min at 95°C, followed by 40 cycles of two-step PCR (denaturation 95 °C for 10 s, annealing and extension 60 °C for 60 s) after 2 min at 50 °C with an initial denaturation step of 10 min at 95 °C. For the detection of *LAT1* mRNA, we used the following oligonucleotides (MWG Biotech): sense primer 5′-CTT CCT CAA GCT CTG GAT CG-3′ and the antisense primer 5′-CCA CGA TGT ACT GCG ATG AA-3′. The same machine settings as described above were used in this case as well. Relative quantification was calculated as published before [17].

### Microarray analysis

To be able to probe for coexpressed genes in the corneal endothelial cells, we used the Affymetrix Gene Chip microarray system (High Wycombe, UK). The cDNA library was obtained as described above for real-time PCR. The microarray analysis was performed by Atlas Biolabs (Berlin, Germany) using the Affymetrix Gene Chip U133 Plus 2.0 according to the manufacturer’s instructions. Data and clustering analyses were determined using Microsoft Excel for Mac, Version 12.1.3 (Microsoft, Redmond, WA) spreadsheets and the online analyzing facilities of the European Bioinformatics Institute/UK [18].

### Proliferation assays

To probe for functional activity of the IDO detected in HCECs, we performed a set of T-cell proliferation assays.

#### Isolation of peripheral blood mononuclear cells from healthy donors

Peripheral blood mononuclear cells (PBMCs) were isolated from heparinized blood by density gradient centrifugation on lymphocyte cell separation medium (Lymphodex; Inno-Train Diagnostik, Kronberg, Germany) [13]. Cells were then washed three times in PBS, and cell viability was assessed by trypan blue.

#### Generation of human monocyte-derived dendritic cells from peripheral blood mononuclear cells of healthy donors

Dendritic cells (DC) were generated according to a standard protocol as previously described [13]. Briefly, PBMCs were isolated from heparinized blood from healthy donors by density gradient centrifugation on lymphocyte cell separation medium (Lymphodex; Inno-Train Diagnostik). After washing, the cells were incubated in Petri dishes (Nunc, Wiesbaden, Germany) at 37 °C and 5% CO_2_ for 90 min and gently washed. The non-adherent T-cells were removed. The adherent monocytes were then cultured in the presence of 1000 U/ml recombinant human interleukin-4 (IL-4; Promocell) and 666 U/ml granulocyte-macrophage colony-stimulating factor (GM-CSF; Sigma-Aldrich Chemie, Taufkirchen, Germany) in RPMI 1640 supplemented with 10% fetal calf serum (FCS), 2 mM L-glutamine (Promocell), and penicillin (100 U/ml)/streptomycin (100 mg/ml; Gibco BRL Life Technologies, Eggenstein, Germany). Cytokines (IL-4 and GM-CSF) were replenished every two to three days by removing half the volume of the medium and adding back the same volume of fresh medium containing cytokines. On day 6 of culture, non-adherent T-cells (immature DCs) were collected by moderate aspiration and seeded into 24 or 96 well plates (Nunc). For maturation, the cells were treated for 48 h with a cytokine cocktail comprising 1 µg/ml prostaglandin E2 (PGE2; Sigma-Aldrich), 1000 U/ml interleukin-6 (R&D Systems, Wiesbaden, Germany), 10 ng/ml TNF-α (Sigma-Aldrich), and 10 ng/ml interleukin-1 (Roche Diagnostics, Mannheim, Germany). The method was used as previously published [19].

#### Direct alloresponse of T-cells toward HCECs

To determine whether the IDO produced by the corneal endothelial cells was functionally active, we set up a direct T-cell proliferation assay. Responder CD4^+^ T-cells (1×10^5^ cells/well) were stimulated with 1×10^4^ irradiated (90 Gray) HCECs in 96 well plates for three days. Proliferation was measured following an 18 h pulse with ^3^H-thymidine (10µl at 5µCi/ml; GE Healthcare, Buckinghamshire, UK).

#### Effect of conditioned HCEC supernatant on T-cell proliferation

To investigate in the L-Kynurenie produced by HCEC following L-Trylpotohane breakdown through IDO induced HCECs could have the power to suppress the activation of cellular immune response.

Unstimulated or stimulated HCECs (with IFN-γ, TNF-α, or a combination of both) were cultured for 72 h, then their supernatants were harvested. A proliferation assay was performed by resuspending target cells (mature DCs) and effector cells (allogeneic PBMCs) with the differently pooled supernatants. Co-culturing of mDCs and allogeneic PBMCs were set up (1:10) in triplicate in 96 well plates in a total volume of 200 µl/well. The plate was incubated for 72 h at 37 °C in a humidified 5% CO_2_ atmosphere. Cell proliferation was measured by an 18 h pulse with ^3^H-thymidine (10 µl ~5 µCi/ml, Amersham Pharmacia Biotech).

Proliferation assays were measured in a beta-counter (Inotech Biosystems, Lansing, MI) with calculation of the counts per minute (cpm) as published before [13]

### ELISA for the detection of TGF-β in tissue culture supernatant

Supernatants were harvested from cells either untreated or treated with IFN-y, TNF-α, or a combination of both as described before. Cells were been cultured for 72 h at 37 °C. We used a sandwich enzyme linked immunosorbent assay (ELISA; TGF-β2 Quantikine ELISA Kit; R&D system) for quantitative determination of TGF-β2 in cell culture supernatants following the manufacturer’s instructions. The optical density of each well was determined using a microplate reader (Tecan) with a wavelength set to 450 nm. All measurements have been done in triplicate.

### Immunohistochemistry

Immunohistochemistry was performed on formalin-fixed and paraffin-embedded rejected human corneal specimens (n=12) with essentially the same protocols and well classified primary antibodies as described in detail previously [20,21]. In addition, a monoclonal antibody directed against IDO (anti-IDO, MAB5412; Chemicon, Hofheim, Germany) was used.

### Apoptosis assay

HCECs were assessed for apoptosis using flow cytometry staining for annexin-V conjugated with fluoresceinisothiocyanate (FITC). The reagents were obtained from BD Biosciences (Heidelberg, Germany) and used following the manufacturer’s instructions. Determination of the apoptosis rate was performed as previously described [22].

### Statistical analysis

Mean values with standard deviation are given for all data. Statistical differences were calculated using a two-tailed and paired *t*-test. All statistical analyses were performed using GraphPad Prism version 4.00 for Windows (GraphPad Software, San Diego, CA). A value of p<0.05 was regarded as statistically significant.

## Results

### IDO, L-kynurenine, and L-tryptophan levels in HCECs

**Figure 2:**
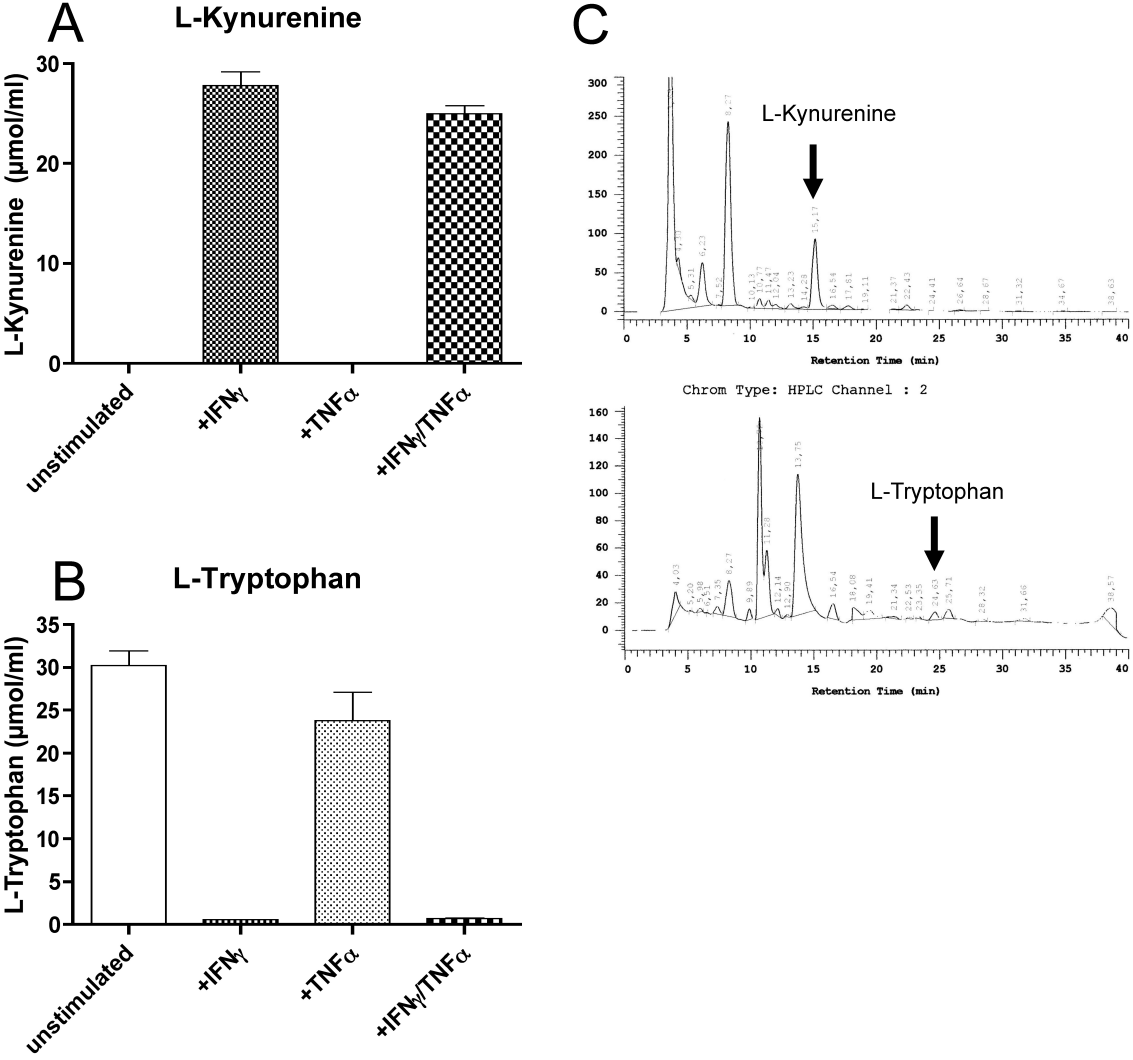
Functional activity of IDO was determined by measuring the concentration of L-kynurenine and L-tryptophan in the tissue culture supernatant of pooled HCECs. HCECs were treated with 500 ng/ml IFN-γ, 100 U/ml TNF-α, 500 ng/ml IFN-γ+100 U/ml TNF-α, or without any of the cytokines. After 72 h, the cell culture supernatants were harvested, and the concentrations of L-kynurenine (**A**) and L-tryptophan (**B**) were detected by HPLC. The difference between the IFN-γ or cocktail stimulated HCECs versus the unstimulated or TNF-α only stimulated HCECs was highly significant (p<0.03). The chromatograms (**C**) show the total free L-kynurenine (top) and L-tryptophan (bottom) of stimulated HCECs. As indicated by arrows, the retention times are different regarding L-kynurenine and L-tryptophan. Interestingly, below L-kynurenine, no further downstream metabolites of the IDO pathway were detected. Furthermore, no L-kynurenine (0.0 µmol) was detected in the unstimulated group or in the TNF-α stimulated group. The bars shown do represent the mean±standard deviation (SD).

To monitor IDO activity in HCECs, we used HPLC to measure the levels of L-tryptophan and its metabolite, L-kynurenine, in the culture supernatants (Figure 2A,B). Cultured HCECs were stimulated with IFN-γ, TNF-α, or a combination of both (IFN-γ/TNF-α). The supernatant of unstimulated and TNF-α-stimulated HCECs showed no L-kynurenine production. In supernatants of HCECs stimulated by IFN-γ and by both IFN-γ and TNF-α, L-kynurenine was found (27.8±1.37 µmol/ml and 25.0±0.8 µmol/ml , respectively; Figure 2A). The difference of L-kynurenine levels between the IFN-γ stimulated or IFN-γ/TNF-α stimulated HCECs and the unstimulated HCECs or TNF-α stimulated HCECs was significant (p<0.03). Highest L-tryptophan levels were found in unstimulated HCECs (30.2±1.8 µmol/ml) and in supernatants of TNF-α stimulated HCECs (23.8±3.4 µmol/ml) while the L-tryptophan concentrations were significantly reduced in the supernatants of IFN-γ stimulated HCECs (0.6±0.04 µmol/ml) and in supernatants of IFN-γ/TNF-α stimulated HCECs (0.7±0.1 µmol/ml; Figure 2B). Again, no statistical differences were found between the IFN-γ/TNF-α and the IFN-γ stimulated group, or between the unstimulated HCECs and the TNF-α stimulated HCECs.

### IDO upregulation in HCECs by cytokine stimulation

To further demonstrate that the L-kynurenine produced by IFN-γ and/or TNF-α and L-tryptophan degradation were caused by IDO, we determined IDO mRNA and protein levels with western blot analysis and RT-PCR.

**Figure 3:**
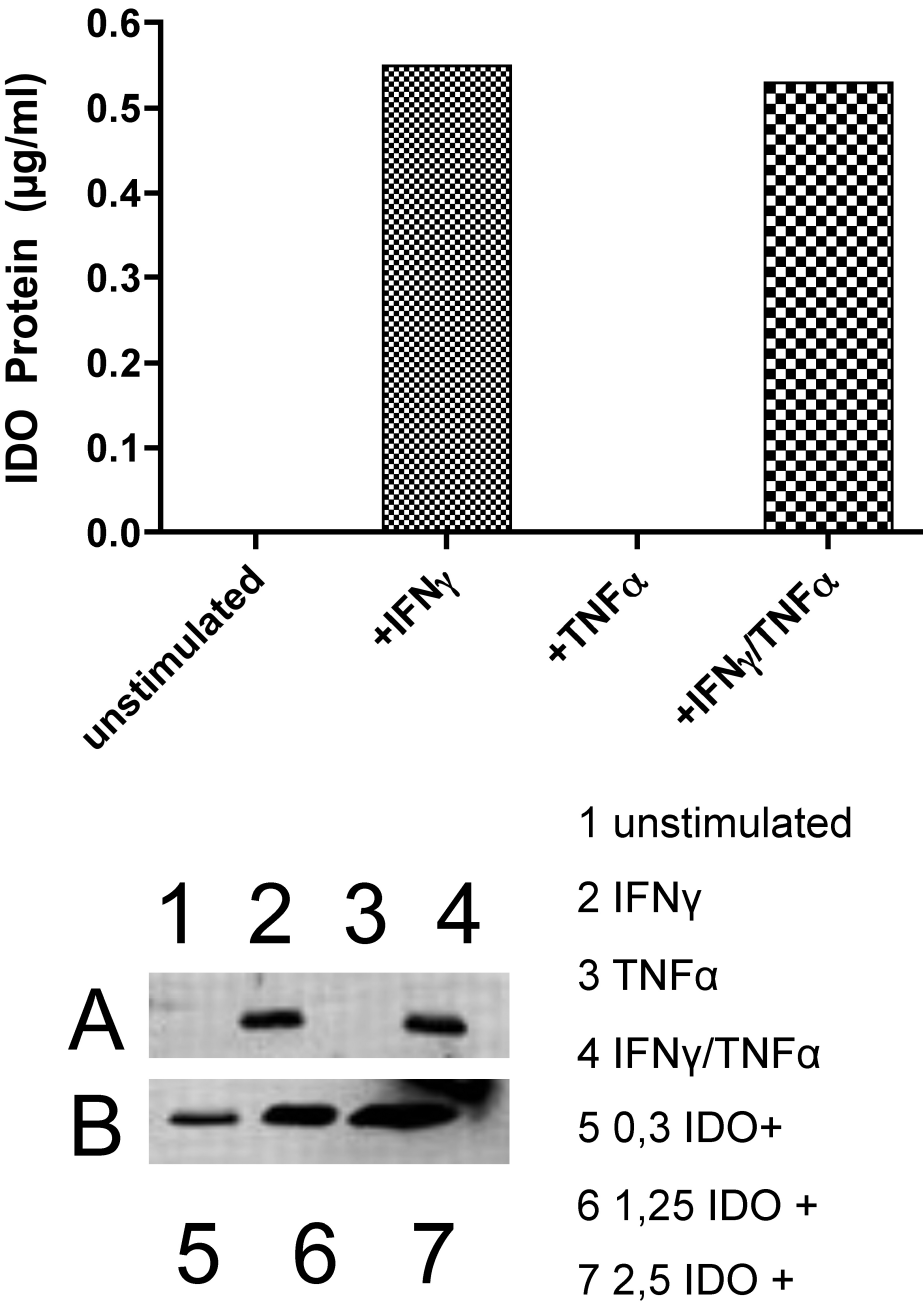
Quantitative determination of IDO protein in HCECs. The top panel shows the quantification of **A**. HCECs were unstimulated (**A**: lane 1) and stimulated with 500 ng/ml IFN-γ (**A**: lane 2), 100 U/ml TNF-α (**A**: lane 3), or IFN-γ+100 U/ml TNF-α (**A**: lane 4) for 72 h. An undetectable protein band was found in unstimulated HCECs (**A**: lane 1) and stimulated HCECs with 100 U/ml TNF-α (**A**: lane 3). Obvious upregulation of protein expression is seen in cytokine stimulation with 500 ng/ml IFN-γ for 72 h (**A**: lane 2) and 500 ng/ml IFN-γ+100 U/ml TNF-α for 72 h (**A**: lane 4). The bottom panel shows the IDO control protein at 0.3 µg (**B**: lane 5), 1.25 µg (**B**: lane 6), and 2.5 µg (**B**: lane 7). For the detection of the bands, protein of three independent experiments has been pooled. Thus, no error bars will be shown.

HCECs were stimulated using the same conditions as described above, and the total protein was extracted. Using SDS-PAGE, western blot analysis, and recombinant IDO protein for the purpose of quantification, IDO protein was analyzed. We were not able to detect IDO in unstimulated or TNF-α stimulated HCECs, but we did detect strong protein expression with western blot in IFN-γ (0.55 µg/ml) and IFN-γ/TNF-α stimulated (0.53 µg/ml) HCECs (Figure 3A,B).

This was followed by a quantitative RT-PCR of the HCECs grown in the same conditions as described above. RNA was extracted and analyzed on a Bioanalyzer (Agilent Technologies, Waldbronn, Germany). For the reverse transcription and generation of the cDNA library, only the RNA with the highest quality was used (Figure 1B). The results shown are normalized against the housekeeping gene, *GAPDH*. Stimulation of HCECs with IFN-γ results in a 112 fold increase of *IDO* expression. An even higher *IDO* expression (160 fold) was seen using the cytokine cocktail of IFN-γ and TNF-α (Figure 1A). The difference between these two groups was statistically significant (p<0.05). In contrast, only a threefold upregulation of *IDO* was detected in HCECs stimulated by only TNF-α. The difference was very significant (p<0.01) when comparing the IFN-γ stimulated HCECs and IFN-γ/TNF-α stimulated HCECs with the unstimulated and TNF-α stimulated cells.

To sum up, HCECs treated with IFN-γ/TNF-α or just IFN-γ showed the highest *IDO* mRNA and protein levels in contrast to untreated HCECs or HCECs treated with only TNF-α.

### T-cell proliferation assays

In the next step, we determined the possible effect of L-tryptophan and IDO activity on T-cell proliferation. IDO degrades L-tryptophan to L-kynurenine, which might induce apoptosis in T-cells. It has to be noted that IDO-dependant apoptosis is independent from the antigen [23]. Highest levels of T-cell proliferation were found in unstimulated HCECs (4478 cpm) and in HCECs stimulated with TNF-α alone (5,439 cpm). The stimulation with IFN-γ or IFN-γ/TNF-α resulted in decreased T-cell proliferation (IFN-γ, 1567 cpm; IFN-γ/TNF-α, 1649 cpm). Again, the difference was statistically significant (p<0.05) when comparing IFN-γ and IFN-γ/TNF-α stimulated cells with the unstimulated and TNF-α stimulated cells. No significant difference was detected between the IFN-γ and IFN-γ/TNF-α stimulated cells.

In the next step we antagonized the IDO effects with 1-methyl Tryptophan (1-MT), which was added to cultured unstimulated (4,000 cpm) and stimulated (IFN-γ, 3,422 cpm; TNF-α, 3,820 cpm; and IFN-γ/TNF-α, 3,649 cpm) HCECs, and performed allogeneic lymphocyte proliferation assays.

The obtained results for IFN-γ and TNF-α/IFN-γ on T-cell proliferation could be attributed to the IDO enzymatic activity. All experiments were performed in triplicate.

### Sandwich ELISA for the detection of TGF-β2 in tissue culture supernatants

To clarify a possible effect of HCEC-derived TGF-β on T-cell proliferation as proposed by the late Streilein group in a murine model [24], we determined the level of TGF-β2 in tissue culture supernatants by performing a sandwich ELISA in unstimulated HCECs and HCECs stimulated with TNF-α, IFN-γ, and a combination of both. TGF-β was neither found in the unstimulated condition, nor in the case of solitary IFN or IFN/TNF stimulated cells. Only a mild TGF-β2 level (7 pg/ml) was seen in the TNF-α stimulated condition (Table 1).

**Table 1:** Detection of TGF-β in tissue culture supernatant*.*

**TGF-β in culture supernantants**	**Units (pg/ml)**
HCECs (unstimulated)	Not detected
HCECs+IFN-γ	Not detected
HCECs+TNF-α	7.0±1
HCECs+IFN-γ+TNF-α	Not detected

Neither in unstimulated nor in IFN-γ or IFN-γ/TNF-α stimulated cells any TGF-β has not been detected in the unstimulated or the IFN-γ or IFN-γ/TNF-α stimulated cells. In contrast, TNF-α stimulated cells showed a mild TGF-β level.

### IDO downstream metabolites generated from HCECs effectively block T-cell proliferation

**Figure 4:**
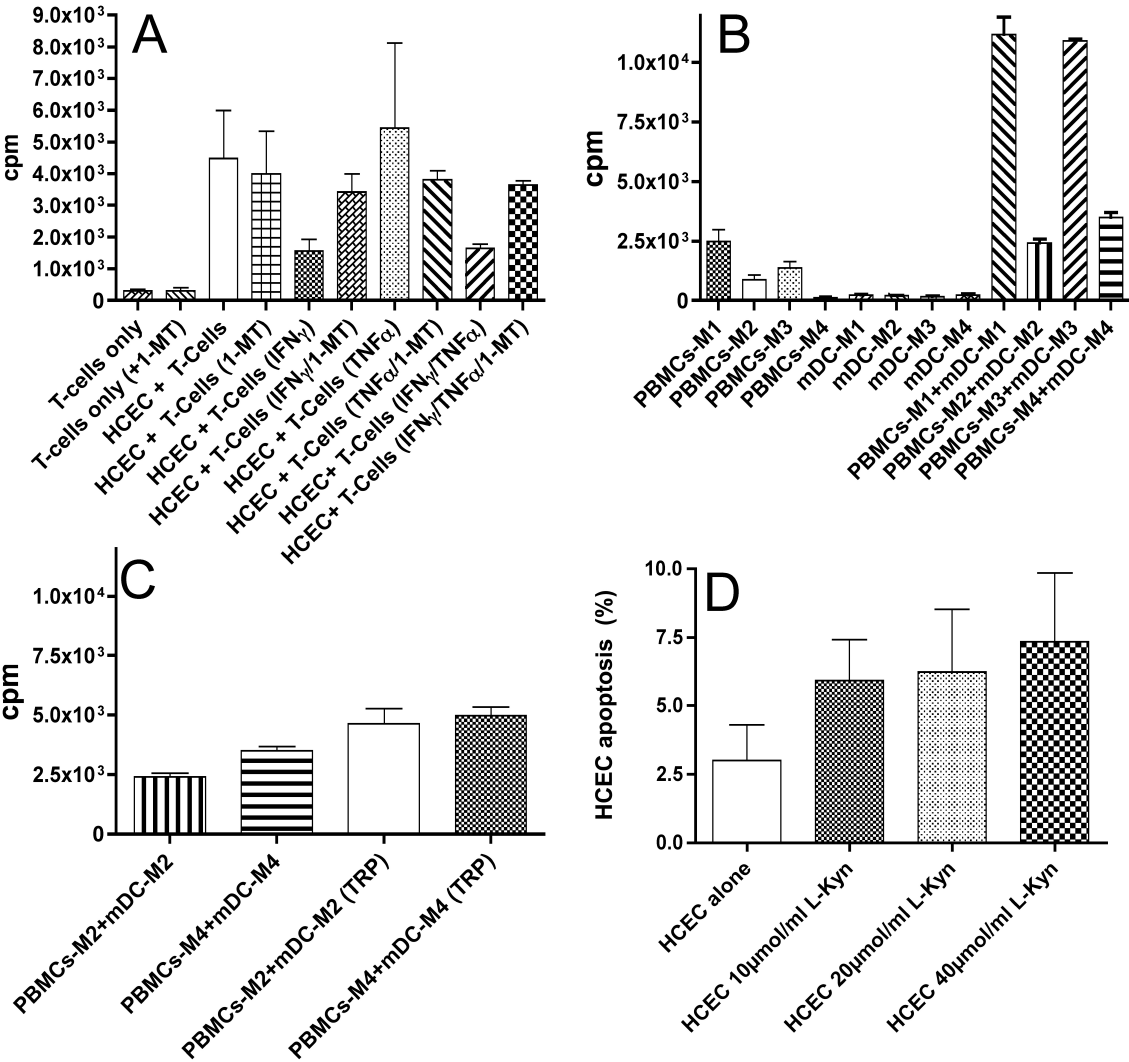
Analysis of conditioned HCECs. **A**: Determination of direct alloresponse of conditioned HCECs toward T-cells is shown. T-cells were either unstimulated or stimulated using IFN-γ or TNF-α or a combination of both. No significant T-cell proliferation could be obtained. HCECs need stimulation of IFN-γ to upregulate class II molecules. As this co-occurred with an upregulation of IDO, a significant induction of T-cell proliferation could not be detected. However, using 1-MT to inhibit IDO, a good proliferation of T-cells up to more than 3×10^3^ cpm was seen (Lane 6 and 10). **B**: The effect of IDO-induced tryptophan metabolites on the proliferation of effector cells (PBMCs) is shown in this panel. Mature DCs were incubated (1×10^4^ cells/well) for 72 h with allogeneic PBMC cells (1×10^5^ cells/well) in three different T-cell culture supernatants of HCECs stimulated with 500 ng/ml IFN-γ, 100 U/ml TNF-α, or 500 ng/ml IFN-γ + 100 U/ml TNF-α. The positive control consisted of culturing PBMC cells with allogeneic DCs in cell culture supernatant of unstimulated HCECs, and the negative control consisted of PBMCs or mDCs alone in different pools of HCECs. T-cell proliferation was determined by ^3^[H]-Thymidin incorporation (cpm) after three days of culturing. We can show that the conditioned medium taken from HCECs stimulated with IFN-γ and IFN-γ/TNF-α led to a significant suppression of T-cell proliferation. **C**: Re-supplementation of the conditioned medium as described in **B** using L-tryptophan is shown. To determine whether the low tryptophan or the produced L-kynurenine was responsible for the reduction in T-cell proliferation, we supplemented the pre-conditioned supernatant to L-tryptophan.  Only a slight yet significant restoration of the T-cell proliferation up to 4×10^3^ cpm could be registered. However, the T-cell proliferation did not reach the level as shown in **B** as L-Kyn was not depleted from the supernatant. **D**: Apoptosis assay using flow cytometry for Annexin V was used to determine dose-dependant toxicity of L-kynurenine toward HCECs. We did see a slight and insignificant increase in apoptosis supplementing the HCEC supernatant to L-kynurenine at 10, 20, and 40 µmol/ml.

To be able to differentiate whether the upregulated IDO itself or the downstream metabolites of the IDO pathway (such as L-kynurenine) together with a low tryptophan medium was responsible for the inhibition of the T-cell proliferation, we used a modified T-cell proliferation assay. An allogeneic T-cell reaction was started as a co-culture of mDCs and T-cells in all cases. We measured the T-cell proliferation by adding the supernatants from HCECs either unstimulated (M1) or stimulated with IFN-γ (M2), TNF-α (M3), or IFN-γ/TNF-α (M4). The IFN-γ (2,425±152 cpm) and the IFN-γ/TNF-α (3,509±181 cpm) supernatants were highly effective in blocking proliferation of the T-cells. However, supernatants from TNF-α treated cells (10,918±69 cpm) and untreated cells M1 (11,187±721cpm) had no effect on T-cell proliferation (Figure 4B).

### Re-supplementation of the conditioned medium using L-tryptophan

To determine whether the low tryptophan or the produced L-kynurenine was responsible for the reduction T-cell proliferation, we supplemented the preconditioned supernatant to L-tryptophan. Only a slight – however significant – restoration of the T-cell proliferation up to 4×10^3^ cpm could be registered as L-Kynurenine (L-Kyn) was not depleted from the supernatant. Thus, T-cell proliferation did not reach the level as shown in Figure 4B (Figure 4C). This gives evidence that both tryptophan deprivation and mainly an increase of L-kynurenine levels would lead to the observed inhibition of T-cell proliferation.

### Apoptosis of HCECs with L-kynurenine

HCECs were assessed for apoptosis using flow cytometry staining for annexin-V. Untreated HCECs would present an apoptosis rate of 3%±1.3%. The addition of 10, 20, and 40 µmol/ml L-kynurenine did slightly extend the apoptosis rate to 5.92%±1.5%, 6.23%±2.3%, and 7.34%±2.5%, respectively (p<0.05). This only shows a minor increase in the apoptosis rate with the rate always below the 7.5% limit, revealing no substantial cytotoxic effect on HCECs at high concentrations (Figure 4D).

### Upregulation of LAT1 transporter, CD74, HLA-DRA, and HLA-DRB1 under proinflammatory conditions

**Figure 5:**
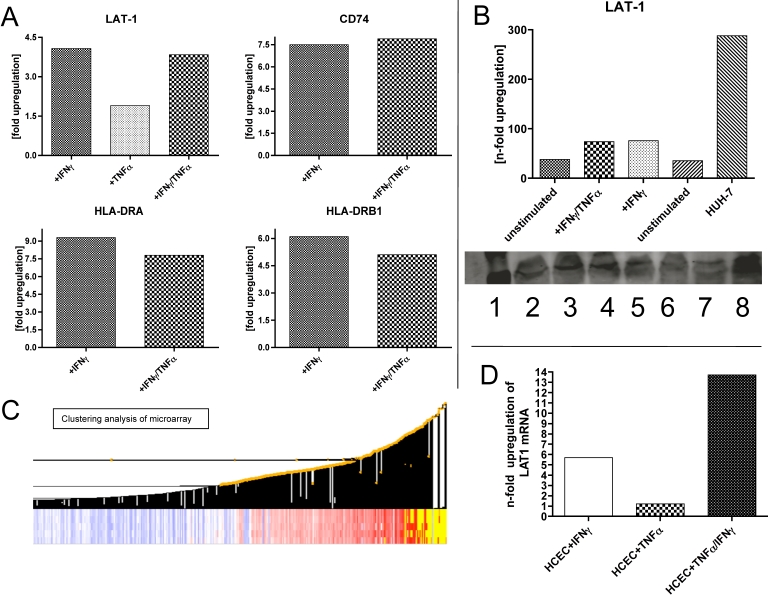
Microarray analysis of HCECs for LAT1 transporter protein, CD74, HLA-DR, and HLA-DRB1 and western blot analysis. **A**: All results shown are normalized against the control (unstimulated HCECs). We could demonstrate a fourfold upregulation of the LAT-1 transporter system upon stimulation with IFN-γ (together with a 2.7 fold upregulation of the class II surface antigens) and a 3.85 fold upregulation following stimulation with the cocktail of IFN-γ and TNF-α. However, the stimulation with TNF-α alone only caused a 1.9 fold upregulation. **B**: Semi-quantitative western blot analysis of LAT1 protein: 1=protein ladder (250 D-10 kDa (Bio-Rad), 2=unstimulated HCECs, 3=HCECs stimulated with IFN-γ, 4=HCECs stimulated with IFN-γ+TNF-α, 5=HCECs stimulated with IFN-γ, TNF-α+Prostaglandin E2 (as a control to antagonize the stimulatory effects of IFN-γ+TNF-α), 6=HCECs stimulated with IFN-γ, TNF-α+Prostaglandin E2+1-MT, 7=HCECs stimulated with TNF-α, 7=HUH-7 liver cell line as control. **C**: The bottom panel shows the exemplary clustering analysis of our Affymetrix assay. **D**: Upregulation of LAT1 following stimulation with IFN-γ, TNF-α and IFN-γ, TNF-α was detected by quantitative real-time PCR. The data shown are normalized against the copy numbers determined from unstimulated HCECs.

As stimulation of HCECs using inflammatory cytokines such as IFN-γ and TNF-α causes upregulation of numerous genes, we used Affymetrix microarray analysis to probe for several genes known to be associated with the IDO metabolic pathway and immunoregulation (Figure 5C). In this setting, we could demonstrate a fourfold upregulation of the LAT1 transporter system upon stimulation with IFN-γ and a 3.85 fold upregulation following stimulation with the cocktail of IFN-γ and TNF-α. However, the stimulation with TNF-α alone only caused a 1.9 fold upregulation. In terms of antigenicity, we found high expression of HLA-DRA and HLA-DRB1 (5 to 6 fold upregulation) following stimulation with IFN-γ but only low expression following stimulation with TNFa. It is important to note that in the murine system, IFN-γ alone was not sufficient to upregulate the expression of class II [25]. Similarly, CD74 (also a member of the HLA-DR family) showed a 7.5 fold upregulation following either IFN-γ or IFN-γ/TNF-α stimulation but no upregulation following only TNF-α stimulation (Figure 5A).

To conclude, the results of our cell culture experiments and T-cell proliferation assays suggest that the IDO found upregulated in HCECs by stimulation with proinflammatory cytokines is biologically active by degrading L-tryptophan to its key metabolite, L-kynurenine, leading to a suppression of T-cell proliferation and potential cytoprotection in HCEC apoptosis.

The upregulation of LAT1 could also be confirmed on the protein level (Figure 5B) and as well on RNA level in a quantitative real-time PCR analysis (Figure 5D). On the mRNA level, we found an upregulation of *LAT1* up to 5.7 fold upon IFN-γ stimulation and 13 fold following the combined stimulation of IFN-γ and TNF-α.

### Immunohistochemistry for IDO and immune cells

We performed immunohistochemistry (IHC) in rejected corneas (n=12). IHC must be regarded as proof of concept as the power is per se limited due to the specimens available. Principally, human tissue can only be obtained either from already completed rejections or from donor grafts that have not been used for transplantation for quality reasons. Different from rejection in animal models, humans will need to be treated immediately and extensively with immunosuppressive drugs such as steroids or cyclosporine, often on a long-term therapy regimen to minimize inflammatory response during subsequent often reversible rejection episodes of varying duration. This seems to be the reason why the corneal full thickness anatomy of the presented histologic specimens appear to be more intact and relatively uneroded by leucocyte infiltration. Furthermore, not all corneal grafts have the same tendency to reject as is the case with keratoconus compared to transplanted grafts into recipient corneas that are inflamed. Accordingly, to test the hypothesis of a possible immunomodulatory contribution of endothelium-derived IDO/kynurenine on maintaining corneal immune privilege, it is obvious to choose the slides with relatively undisturbed endothelial histology due to their lower propensity for rejection.

**Figure 6:**
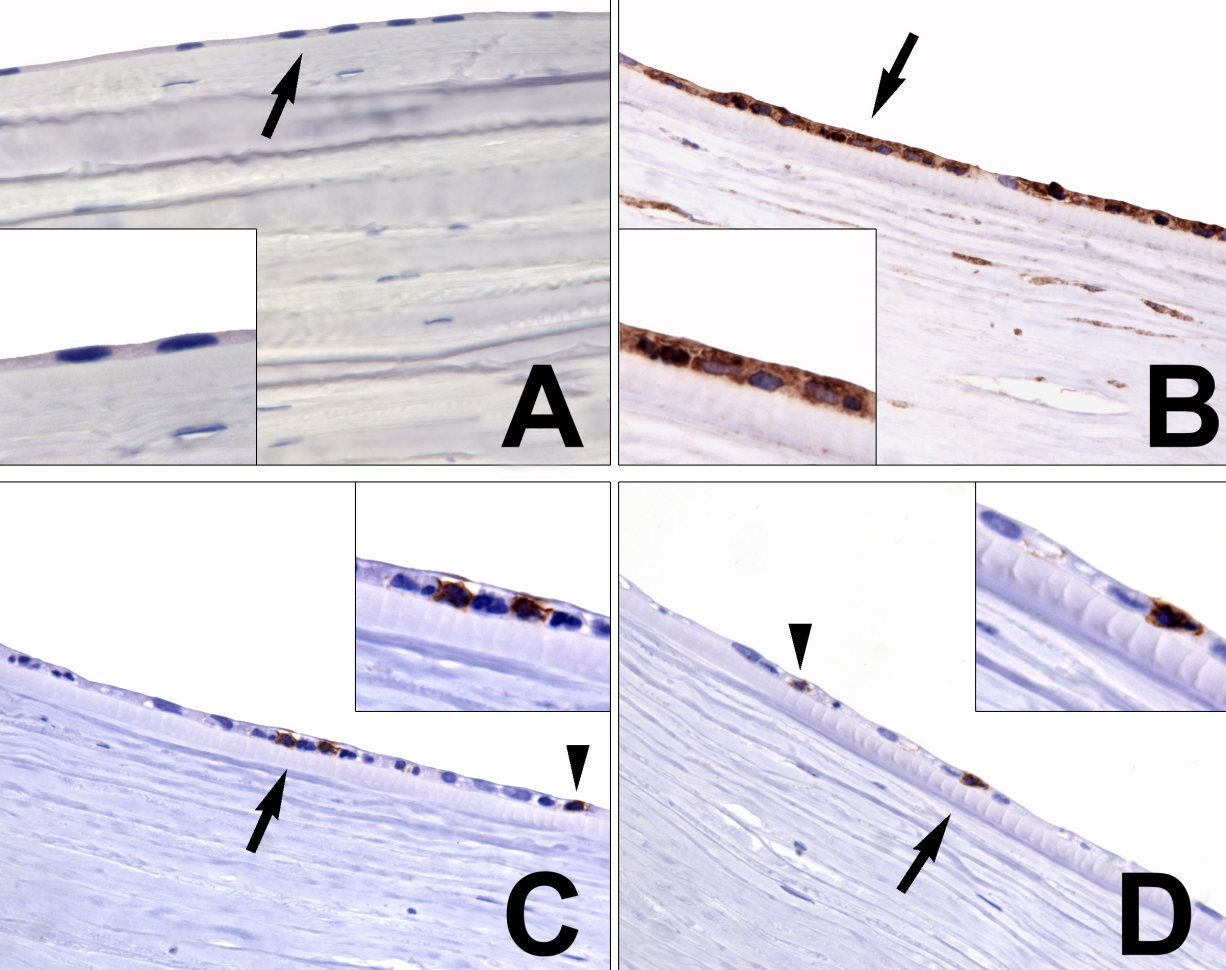
Immunohistochemistry for IDO and CD3+ and CD8+ lymphocytes in the corneal endothelium. **A**-**B**: IDO; **C**: CD3+ T – lymphocytes; **D**: CD8+ T - lymphocytes. Magnification **A**-**D** is 400X; inlays in **A**-**D** represent twofold enlarged details from respective images marked by arrows. **A**: non-rejected cornea negative for IDO (**A**) and no T-cell infiltration (data not shown); **B**-**D**: rejected cornea transplant positive for IDO (**B**) with infiltration of CD3 + T-lymphocytes (**C**; arrow, arrowhead) and CD8+ T-lymphocytes (**D**; arrow and arrowhead) within IDO-positive (**B**) cornea. **B**-**D** are images from serial sections.

The pattern of IDO expression varied. Rejected corneal transplants showed high IDO expression. IDO was found in the corneal endothelial cells or in the corneal epithelial layer, depending on the underlying corneal disease and predominant site of immune reaction. In contrast, IDO was found to be absent or only scantly expressed in the non-rejected controls (Figure 6A-D).

## Discussion

The kinetics of corneal graft failure is different from those of vascularized organ grafts [4]. Generally, corneal allotransplantation is highly successful in the short term outcome with graft survival rates of 90% one year after surgery [2]. In most cases, immunosuppression is limited to the topical application of corticosteroids. As HLA class I and class II-DR matching between donor and recipient had no convincing benefit for graft survival, it is not routinely performed [2]. The cornea is regarded to be an immunologically privileged site, which might prolong corneal graft survival, due to the lack of vessels and immunologic properties of the corneal endothelial, stroma, and epithelium [4].

In the context of corneal allograft, the endothelial monolayer in direct juxtaposition with the anterior chamber constitutes the vulnerable target of an attack by intraocular immune effector cells during rejection episodes [26]. Even though the corneal endothelium is only a single layer in thickness and expresses only scant quantities of MHC antigens, it is vulnerable to immunological attack [27]. Among the multiple and redundant mechanisms that operate in the effector phase of corneal graft rejection, the T-cell mediated response is considered to be of the most important cause of graft loss following rejections. In addition, corneal endothelial cells have distinct molecular strategies to reduce their antigenic visibility to CD4^+^ and CD8^+^ effector T-cells and to alter the functional program of responding T-cells. As part of the corneal immune privilege, it is known to have a unique pattern of MHC class I and II expression [5,28]. For example, in the murine system, IFN-γ is not sufficient to upregulate class II expression, but a cocktail of cytokines is sufficient to do so [25]. Although MHC class I antigen expression on the murine corneal endothelium is known to be weak, the endothelial layer is functionally immunogenic and antigenic for allospecific cytotoxic T-lymphocyte (CTL) responses. That is, allogeneic corneal endothelium stimulates robust CTL responses in vivo and is susceptible to CTL-mediated lysis in vitro [29,30].

In our human system, however, we found that IFN-γ is sufficient to stimulate class II expression as previously seen in vascular endothelial and epithelial cells [31]. However, at this stage, it is unclear why there is a fundamental difference between the human and mouse system.

Several reports have demonstrated IFN-γ mediated upregulation of IDO in the murine system [32,33]. This has been shown to have an anti-inflammatory effect in various models including corneal transplantation [8]. Our data indicate that HCECs can constitutively express immunoreactive IDO protein.

Interestingly, in the set-up without any IFN-γ and/or TNF-α stimulation, there is no biologically active IDO as determined by an assay for L-kynurenine (Figure 2). IDO from unstimulated cells were only found on the RNA level but not on the protein level and thus were not found potent enough to result in a significant inhibition of T-cell proliferation. However, upon stimulation using proinflammatory cytokines, T-cell proliferation could be inhibited. This effect could be reversed by inhibiting upregulated IDO using 1-MT. The human endogenous IDO activity following an immunologic attack of the corneal endothelium represents an innate mechanism of HCECs for immunological modulation as previously shown in a mouse model [8].

More significantly, using microarray analysis (Figure 5), we demonstrate that an L-amino acid transporter protein is upregulated in HCECs by inflammatory cytokines. This transporter is known to exchange tryptophan for kynurenine, thereby coupling tryptophan starvation in the T-cell microenvironment with kynurenine-induced T-cell inhibition (Figure 4B,C). Conversely, IDO-producing HCECs that are expressing the LAT-transporter system mostly maintain their cell viability, only showing a slight apoptotic increase after accumulated exposition to L-kynurenine (Figure 4D). We attribute this to the strict counter-exchange of tryptophan and the IDO downstream metabolic products (L-kynurenine).

Nevertheless, the question arises whether the kynurenine levels found to be suppressive in our cell cultures reflect the concentrations at the site of production in vivo [5,34]. As a matter of fact, the low serum blood levels of the tryptophan catabolite, kynurenine (1–3 μmol), do not necessarily exclude the existence of high levels at defined sites of the body. This has previously been evidenced in the placenta or murine cornea, thereby creating a local site of immunosuppression toward fetal tissue during mammalian gestation or grafted tissue, respectively [35].

In addition, the basal level of tryptophan in the serum is mainly controlled by the homeostatic enzyme, IDO (it’s expression is mainly confined to the liver and is not induced or regulated by signals from the immune system). In contrast, the inducible expression of IDO is subject to complex regulation by an array of immunologic signals including IFN-γ and TNF-α and can be induced by such signals in various cell types including HCECs (Figure 4) [8,36]. Applying this phenomenon to in vivo conditions would implicate that relatively low kynurenine concentrations may be sufficient for the suppression of the T-cell response due to the fact that under the condition of human corneal graft failure, the exposure time is obviously markedly longer than the duration of cell cultures used in the present study. Hence, as part of a relevant biological property of HCECs, significantly lower kynurenine concentrations might be effective in their T-cell inhibitory potential at concentrations that probably could be achieved in the extracellular microenvironment while at the same time preserving HCEC viability (Figure 4D) [8,36].

So our model must make the assumption that the tissue microenvironment that surrounds the grafted tissue is not in equilibrium with the blood and that those local concentrations of tryptophan or its cytotoxic catabolites can vary markedly from that observed in the plasma. Given that previous mouse studies [8] indicate that IDO-overexpressing corneal endothelial cells (CECs) can induce immunosuppression in vivo, the admittedly artificial culture model might nevertheless yield clinically relevant insights. It is therefore conceivable that the tryptophan-kynurenine exchange by this strictly local cycle presented in our study on HCEC may account for an endogenous immunosuppressive mechanism during allograft rejection comparable to the previously observed murine model of allograft rejection [8]. This may operate as part of a negative feedback loop to limit local immune responses underlying ocular immune privilege by supporting the two-pronged inactivation of neighboring T-cells. By depriving T-cells of the local tryptophan pool and increasing the kynurenine concentrations, a self-limiting immune protection mechanism for resting postmitotic HCECs might be constituted (Figure 4). As Th1 CD4^+^ T lymphocytes are selectively sensitive to IDO-mediated tryptophan deprivation [37] in HCECs, the following L-kynurenine-dependent block of cell cycle progression of these cells might ultimately prolong survival of grafted corneal tissue [8].

If this is functional in vivo in the human system, the mechanism delineated by our findings might have far reaching consequences for immunoregulation and for the pathogenesis of corneal graft failure. The ability of the cornea to promote immunologic unresponsiveness by deflecting the systemic immune response away from a Th1 pathway might be an integral component of the immune privilege of corneal allografts during proinflammatory environment. This may represent a novel adaptation by the anterior chamber to preserve vision by avoiding immunogenic inflammation in the effective suppression of donor antigen-reactive Th1 activity. Recently, it has also been shown that constitutive expression of CD95L on the corneal endothelium is critical to its immune privileged status by triggering apoptosis in naïve alloreactive T-cells. Accordingly, when grafted to a heterotopic site and in the absence of the corneal epithelium, CD95L prevents allosensitization and renders murine endothelial cells and stroma resistant to immune destruction following allograft rejection directed at MHC alloantigens. Full thickness allogeneic corneas, however, were rejected acutely due to the overpowering epithelial immunogenicity [5,28]. The presence of CD95L in the human corneal endothelium is already well known [38,39].

Our current human model expand the survey of the corneal immune privilege by demonstrating that effective inhibition of T-cell proliferation depends additionally on rapid delivery of the cytotoxic tryptophan metabolites (L-kynurenine) to the local environment of HCECs (following adequate upregulation of IDO).  This is likely to be the case in rejecting corneal tissue thereby comprising an in vivo mechanism of maintaining ocular immune privilege in the anterior chamber (Figure 6). In this case, the HCECs do not only rely on passive mechanisms but also upregulates an active carrier system (LAT1) for the transport of tryptophan inside the cell and for the delivery of the cytotoxic metabolites to the outside compartment in an attempt to limit the amount of T-cell induced corneal tissue rejection.

If the human immune system utilizes IDO-producing HCECs for suppression of deleterious immune reactions as a means of active down-regulation of Th1 immune responses to alloantigenic cells introduced into the anterior chamber of the eye, then therapeutic induction of IDO, namely the possibility that IDO transfection of tissue allografts might lead to a reinforcement of this innate mechanism.  In this setting the induction of tolerance in a human transplant might be attempted. As a result, corneal rejection episodes could possibly be delayed or prevented as has already been demonstrated in murine corneal transplants [8]. Therefore, manipulating immune responses to improve clinical outcomes on account of the limited accessibility of donor grafts worldwide is an important challenge in improving organ/tissue transplantation rates. Whether IDO need to be delivered to the entire graft or just to a population of tolerogenic antigen presenting cells (APCs) requires further investigation.
